# Impact of the COVID-19 Pandemic on the Diagnosis and Prognosis of Melanoma

**DOI:** 10.3390/jcm11144181

**Published:** 2022-07-19

**Authors:** Antonio Martinez-Lopez, Pablo Diaz-Calvillo, Carlos Cuenca-Barrales, Trinidad Montero-Vilchez, Manuel Sanchez-Diaz, Agustin Buendia-Eisman, Salvador Arias-Santiago

**Affiliations:** 1 Dermatology Unit, Virgen de las Nieves University Hospital, 18014 Granada, Spain; pdc.muro@gmail.com (P.D.-C.); carloscuenca1991@gmail.com (C.C.-B.); tmonterov@gmail.com (T.M.-V.); manolo.94.sanchez@gmail.com (M.S.-D.); salvadorarias@ugr.es (S.A.-S.); 2 TECe19-Investigational and Traslational Dermatology Research Group, Instituto de Investigación Biosanitaria (IBS), 18012 Granada, Spain; abuendia@ugr.es; 3 Department of Dermatology, University of Granada, 18011 Granada, Spain

**Keywords:** melanoma, COVID-19, SARS-CoV-2, pandemic

## Abstract

Background: Early detection of melanoma is one of the main diagnostic goals of dermatologists worldwide, due to the increasing incidence of the disease in our environment. However, the irruption of the SARS-CoV-2 pandemic has posed a challenge to global healthcare, forcing systems to focus their resources on the fight against COVID-19. Methods: Retrospective cohort study. The exposed cohort were patients diagnosed with melanoma in the year after the general confinement in Spain (15 March 2020) and the unexposed cohort were patients with melanoma diagnosed in the previous year. Results: 130 patients were included. No differences were observed between demographic characteristics in both cohorts. The mean Breslow of melanoma before the onset of the pandemic was 1.08, increasing to 2.65 in the year after the onset of the pandemic (*p* < 0.001). On the other hand, the percentage of melanomas in situ decreased from 38.96% to 16.98% in the year after the declaration of the state of alarm in Spain. Conclusions: The SARS-CoV-2 outbreak has led to a reduction in the early diagnosis of melanoma, with an increase in invasive melanomas with poor prognosis histological factors. This could lead to an increase in melanoma-related mortality in the coming years in our environment.

## 1. Introduction

The diagnosis and treatment of malignant tumor conditions is one of the main health care activities in the dermatology practice. A recent study carried out in the Spanish population estimates that 18.5% of dermatological diagnoses correspond to malignant tumors, placing malignant melanocytic lesions as the third cause of dermatological malignancies [1]. The incidence of this tumor is progressively increasing from the middle of the last century to date. Thus, a recent European-based study has shown an increase of between 4 and 4.8% per year in the incidence rates of melanoma in Western countries over the last 70 years, thus reflecting the increase in the incidence of this neoplasm in our environment [2]. In Spain, a meta-analysis published in 2016 showed an incidence rate of 8.76/100,000 person-years using all studies and 9.72/100,000 person-years including only the most recent studies. These rates, despite being lower than those of other European countries, also show a progressive increase in the incidence of melanoma in the last years in Spain [3]. Both studies have shown an increasing mortality rate for melanoma in recent years, and it is expected that in our environment, unlike other European countries, this rate will continue to increase due to the progressive aging of the population and the worse biological behavior of melanoma in the elderly patient [3,4].

In December 2019, in Wuhan (China), a new virus called severe acute coronavirus type-2 (SARS-CoV-2) causing COVID-19 disease appeared. This virus spread rapidly throughout the world and, to date, has produced more than 400 million cases and caused more than 4 million deaths worldwide [5]. Several models have supported health system managers in predicting the impact of the pandemic in different periods, with a good correlation between expected and observed cases. Thus, models combining fuzzy logic with fractal theory have obtained up to 98% accuracy [6,7]. This disease has forced changes in the lifestyle of patients, as a result of the lockdown and quarantines imposed in the most affected countries [8,9]. In addition, these measures have also affected the different health systems, which have had to employ different strategies in order to maintain the diagnosis and follow-up of their patients. In dermatology, the use of teleconsultation and teledermatology (sometimes followed by teledermoscopy) has been extended during the worst stages of the outbreak, making it possible to maintain non-face-to-face care activities while avoiding patient visits [10,11].

However, despite the great usefulness of this tool for the early detection of skin tumors, its use is not free of the risk of underdiagnosing melanomas. For example, although teledermatology with teledermoscopy allows an accurate diagnosis of pigmented lesions, it is still less accurate than face-to-face dermatologic consultations, as it does not allow a complete body examination of patients. Moreover, whereas teledermatology allows health care to be provided in areas with a shortage of dermatologists, such as some rural areas, it requires the extension of certain technical means, such as high-speed Internet lines, dermoscopes and adapted cameras, which are not available in all centers. On the other hand, virtual consultations greatly reduce one of the essential activities of dermatologists, which is the communication of primary prevention and health promotion messages about the need for sun protection and knowledge of the warning signs of pigmented lesions so that the patient can detect a tumor lesion early [12,13,14]. However, teledermatology also has important advantages from the point of view of diagnostic prioritization. Thus, it allows early diagnosis and treatment of melanomas referred by this route even in less than 24 h from the visualization of the images. Furthermore, it makes it easier to avoid unnecessary trips for follow-up consultations, as well as allowing the indication of complementary tests before the face-to-face consultation [11]. In addition, a recent study has shown patient satisfaction with the use of this tool during the COVID-19 pandemic, highlighting the speed and efficiency in receiving a diagnosis [15].

**Figure 1:**
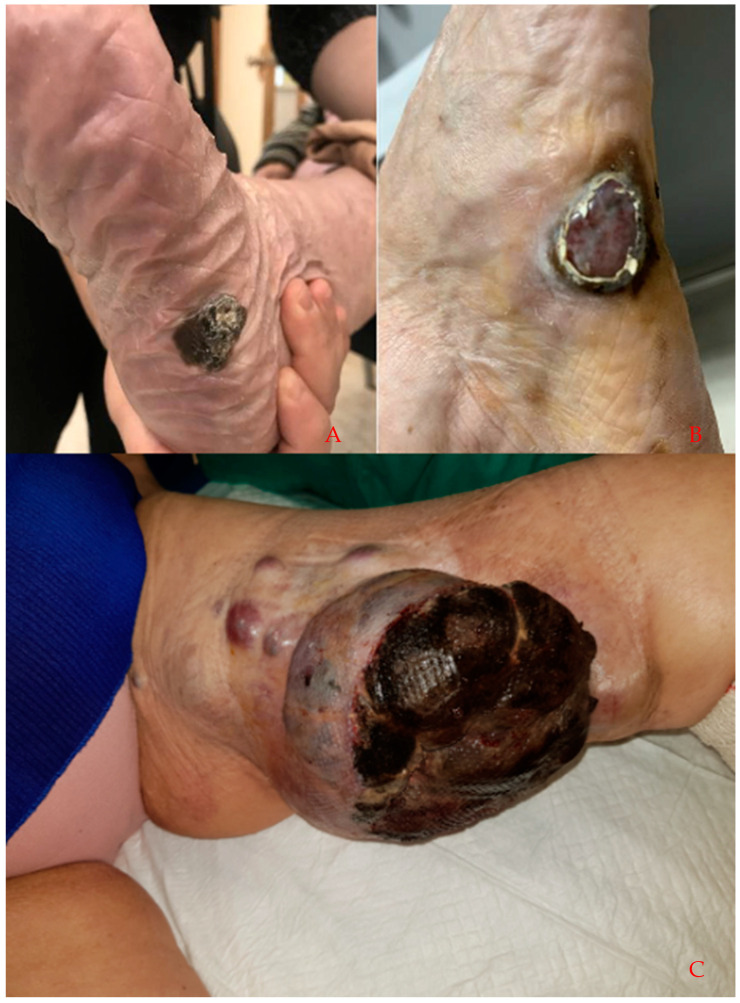
Clinical images of malignant melanoma diagnosed after the onset of the COVID-19 pandemic. (**A**) 81-year-old female patient with a 3.2, nonulcerated melanoma. (**B**) 65-year-old male patient with a 4.1, ulcerated melanoma. (**C**) 54-year-old female patient with a locally advanced melanoma.

Despite most dermatology departments having adapted to maintain the clinical and surgical care of patients with urgent and preferential dermatologic pathology, such as patients with melanoma and advanced non-melanoma skin cancer, from March 2020 to the end of that year, a reduction of between 16% and 77% in the number of consultations received by different dermatology departments worldwide has been observed [16,17,18]. This reduction in consultations may be partly due to the perception that face-to-face dermatology consultations could be a vector of SARS-CoV-2 transmission [19]. This may have led to a lower diagnosis of melanomas since the beginning of the pandemic. In addition, the delay in diagnosis may have led to an increase in the number of large and/or advanced melanomas at the time of diagnosis compared with the situation prior to the outbreak of COVID-19 (Figure 1). With all this background, the aim of this study is to evaluate the impact of the COVID-19 pandemic on the diagnosis, staging and prognosis of melanomas, comparing the data obtained in patients diagnosed with melanoma in the year after the onset of general confinement with those obtained in the same months of the previous year. On the other hand, the study wishes to evaluate whether this possible diagnostic delay could lead to a reduction in the survival of patients diagnosed with melanoma after the onset of the pandemic.

## 2. Materials and Methods

A retrospective cohort study was performed collecting data on patients diagnosed with melanoma in the 12 months prior to the establishment of the first general confinement via alarm status in Spain (15 March 2020) and in the 12 months thereafter. The selection of this period is based on the fact that, after 15 March 2020, a general 8-week lockdown was imposed in Spain in which all non-essential activities were suppressed and most primary care centers were closed to provide support to patients with COVID-19, with the majority of consultations being carried out by telephone. In the remaining waves of the pandemic, these restrictions on health care access were not imposed. Data collection was performed in December 2021 using the database of the Melanoma Unit of our center. All patients included were diagnosed and followed up at the Melanoma Unit of the Hospital Universitario Virgen de las Nieves in Granada, Spain. The study was approved by the Ethical Committee of the Hospital Universitario Virgen de las Nieves. The inclusion criteria were, as follows:

- Adult patients diagnosed with melanoma through histological study in the established study periods.
- Complete staging through clinical evaluation, imaging tests and/or sentinel lymph node biopsy (in cases that were indicated following the AJCC 2018 indications).
- At least one follow-up visit at the Melanoma Unit of the Hospital Universitario Virgen de las Nieves.

Patients who did not meet these criteria were excluded from the study. Epidemiological data on age and sex, time of diagnosis (pre- or post- pandemic onset), histological data of melanoma (Breslow, ulceration, mitosis, microsatellite, lymphovascular invasion and perineural invasion), data on disease progression (development of lymph node, in-transit or systemic metastases), TNM staging and tumor staging were collected in all patients. In-transit metastases are defined as those melanoma metastases located between the primary tumor and the lymph drainage region. Perineural invasion was considered positive when tumors invading nerve fibers larger than 0.1 mm were observed.

A descriptive analysis of the data was performed, with means, medians, and standard deviations for quantitative variables and percentages for qualitative variables, in order to show the characteristics of the sample. On the other hand, to evaluate the possible reduction in 5- and 10-year survival of patients diagnosed with melanoma after the onset of the pandemic, an estimation of the survival was performed taking as a reference the data reflected in the AJCC 2018 guideline [20]. To assess the normality of the variables, the Saphiro–Wilk test was used. After observing that the continuous variables included did not follow a normal distribution, a univariate analysis of the different variables was performed using nonparametric tests (Wilcoxon test). Those variables that had a relevant association with the date of melanoma diagnosis were included in a multivariate analysis. Contingency tables were used to compare the quantitative variables and the chi-square test or Fisher’s exact test was used when necessary. Moreover, a Kaplan-Meier analysis was conducted in order to evaluate the effect of the diagnosis period on the estimated 5-year and 10-year survival. Those differences that presented a *p*-value < 0.05 were accepted as statistically significant. JMP 14.1 software (SAS Institute, Cary, NC, USA) was used for the statistical analysis.

## 3. Results

130 patients were included, 77 were diagnosed with melanoma before the onset of the outbreak and 53 were diagnosed subsequently, representing an 18.46% reduction in annual melanoma diagnoses in the 12 months after the onset of confinement. No significant differences were observed in the age and sex of patients before and after the onset of the pandemic. Regarding the histological characteristics of melanoma (Table 1), the univariate analysis showed that the mean Breslow thickness of the patients prior to the onset of confinement was 1.08, increasing to 2.65 in the year after confinement (*p* < 0.001). Before the onset of the pandemic, 11.69% of melanomas were ulcerated, a poor prognostic feature that increased to 22.64% after the onset of confinement, although this difference did not reach statistical significance (*p* = 0.098). In relation to the presence of mitoses, the mean number of mitoses of the melanomas included prior to the pandemic was 1.40, a value that increased to 3.58 in the subsequent year (*p* = 0.016). No significant differences were observed with respect to the presence of microsatellites and lymphovascular invasion, although significant differences were observed in the presence of perineural invasion. Regarding tumor staging, before the onset of the pandemic 38.96% of melanomas were in situ, a percentage that decreased to 16.98% in the year after the onset of confinement (*p* = 0.005). On the other hand, the percentage of stage II and III melanomas in the year prior to confinement was 11.69% and 10.39%, respectively. The percentage of these stages one year after the declaration of the general lockdown was 22.64% and 20.75%, respectively. A multivariate analysis was conducted in order to observe the effect of the diagnosis period on the relevant variables noted in the univariate analysis. Multivariate analysis showed that patients diagnosed after the pandemic had a higher Breslow thickness (Log 1.505, *p* = 0.031) independently of the effect of the other variables.

**Figure 2:**
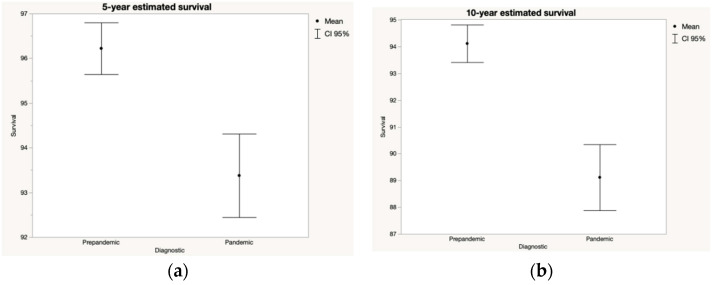
5-year (**a**) and 10-year (**b**) estimated survival according to AJCC 2018 staging system of the patients with malignant melanoma that were diagnosed before and after the onset of the SARS-CoV-2 pandemic. Considering tumor staging, a lower mid-term and long-term survival of patients diagnosed with melanoma after the onset of lockdown might be observed.

**Figure 3:**
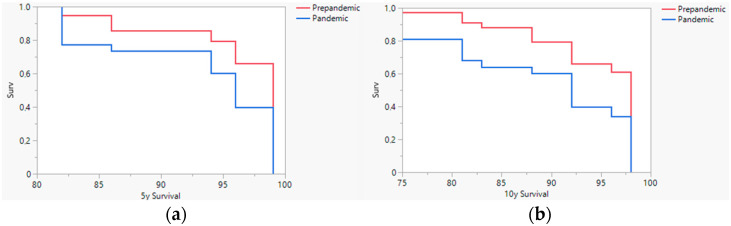
5-year (**a**) and 10-year (**b**) Kaplan-Meier survival analysis. The estimated survival analysis showed worse estimated survival in patients diagnosed after the pandemic.

Concerning the estimated 5- and 10-year survival of patients diagnosed before and after the pandemic (Figure 2), considering the tumor staging at diagnosis, there was a statistically significant worsening in the prognosis of patients diagnosed after March 2020. Thus, the estimated 5-year survival of the melanomas diagnosed after the lockdown could be decreased by 3%, with the estimated 10-year survival rate going from 94% to 89% before and after the onset of the pandemic, respectively. Kaplan Meier analysis showed that the differences in 5-year and 10-year estimated survival were statistically significative (*p* < 0.001) (Figure 3).

## 4. Discussion

In our study, we have observed an increase in the Breslow thickness of melanomas detected in our center the year after the declaration of general confinement in Spain regarding the previous year. In addition, we have noted an increase in certain characteristics of poor tumor prognosis such as the presence of mitosis, as well as a reduction in melanomas in situ and an increase in stage II and III melanomas with respect to the year prior to the onset of the pandemic.

The development of the SARS-CoV-2 pandemic has had a significant impact on both the diagnosis of new tumors and their prognosis [21]. Several studies have shown a significant reduction in the diagnosis of new melanomas following the spread of COVID-19. A recent observational study performed in the Italian population showed a reduction in melanoma incidence in the period March–October 2020 compared to the same period in 2019 (28.7 vs. 32.8). This study also revealed that the main reduction in incidence occurred between the months of March and April 2020 [22]. On the other hand, other studies have also reported a significant reduction in the incidence and diagnosis of new melanomas in 2020 over previous years, in line with the results obtained in our study [23,24,25]. A possible explanation for this reduction in diagnoses may be due to patients’ fear of consulting during the worst months of the pandemic and potentially spreading the disease to family members due to the close contact derived from a face-to-face medical consultation [26].

The presence of high-risk and poor prognostic factors for melanoma progression, such as Breslow thickness, ulceration, or number of mitoses, has also been affected by the SARS-CoV-2 pandemic. Different groups have published similar studies providing data on the impact of the pandemic on different clinical and pathologic melanoma characteristics. A recent Italian multicenter study evaluated melanomas excised within two months after the end of the lockdown, finding a higher Breslow thickness as well as a higher number of ulcerated melanomas and with a large number of mitoses, especially in melanomas excised in northern Italy [27]. In our study, we have observed an increase in Breslow thickness in melanomas diagnosed in the year after general confinement relative to the previous year, as well as an increase in the number of mitoses and in the percentage of ulcerated melanomas. The difference between our approach and the Italian study is that we wanted to evaluate a more extended period and to compare it with the same period of the previous year. A recent study carried out in the Austrian population has not observed differences in Breslow in the year after confinement, although statistically significant differences in the presence of ulceration have been observed [28]. Other studies carried out in the Spanish and American populations have shown a significant increase in Breslow thickness after the onset of the pandemic. Both studies have also reported an increase in the diagnosis of thick melanomas. In addition, the American study has revealed an increase in the number of mitoses and satellites in the period studied after the declaration of the SARS-CoV-2 pandemic [29,30]. The possible difference between the data reported in different countries may be due to the non-uniform magnitude of the pandemic waves throughout the world, and the results may be influenced by the period analyzed in each series. Van Not et al., in a Dutch-based series, have reported a significant fluctuation of the observed/expected melanoma ratio during the pandemic waves [31]. Late diagnosis of melanoma was also reflected in the worse tumor staging of patients diagnosed after the onset of the pandemic. The reduction in the percentage of in situ melanoma diagnosis and the increase in invasive melanoma diagnostic from March 2020 observed in our study is in agreement with what has been published by other authors [25,29,30].

This increase in poor prognostic factors for melanoma after the onset of the SARS-CoV-2 pandemic has led to a significant increase in the diagnosis of locoregionally advanced melanomas (stage III), either through diagnosis of a positive sentinel lymph node biopsy or through the diagnosis of lymph node metastases, in transit or satellites. Thus, in our study we have found a significant increase in melanomas diagnosed at stage III in the post-COVID period, results in line with those previously published by other authors [32].

Considering all these facts, a worse prognosis of melanoma patients diagnosed after the general confinements caused by the pandemic could be expected. In our study, we have observed a statistically significant reduction in the estimated 5- and 10-year survival of patients diagnosed after March 2020. A possible explanation for this may be due to the worsening of the histopathological characteristics of melanomas with the delay in their diagnosis and treatment. Thus, some Spanish authors have estimated a reduction in survival at 5 years of 1.9% and at 10 years of 2.4% in those melanomas whose diagnosis is delayed by 3 months [33]. In this line, a recent study estimates a 7% reduction in survival rates in patients with melanoma diagnosed after March 2020 [34]. These results are only an initial estimation of the possible effect of the outbreak on the survival of patients with melanoma, and large global melanoma registries will have to show the real effect of the pandemic on long-term survival.

Our study is subject to several limitations. Firstly, those inherent to retrospective studies, in which there may be information not collected on patients seen during both periods. In addition, as this is a single-center study, the sample is limited, which may make it difficult to achieve statistical significance in some of the variables studied.

## 5. Conclusions

In this study, we have observed a significant reduction in the number of melanomas diagnosed in the year following the onset of general confinement due to the SARS-CoV-2 pandemic regarding the previous year. In addition, we have also noted an increase in poor prognostic factors in these patients, such as an increase in Breslow thickness, ulceration or the presence of advanced stages at diagnosis. All these facts show the effect that the pandemic has had on the decrease in the early diagnosis of melanoma, which could lead to a reduction in the long-term survival of patients diagnosed with melanoma. Multiple national and international series have been published with heterogeneous data but overall they seem to show worse histologic and prognostic characteristics of patients diagnosed with melanoma after the onset of the SARS-CoV-2 pandemic. Thus, systematic reviews and meta-analyses that comprehensively analyze the available data worldwide will be necessary.

## Figures and Tables

**Table 1:** General characteristics of the melanoma patients before and after the onset of the SARS-CoV-2 pandemic. No differences were observed between age and sex in the two study groups. As shown in the table, patients diagnosed with melanoma after the onset of the pandemic had a higher Breslow, a higher number of mitoses and a higher percentage of patients presented with tumors with perineural invasion. There was also a trend in these patients to have more ulcerated tumors.

	Pre-Pandemic (*n* = 77)	Pandemic (*n* = 53)	*p*
Age	63.31 +/− 1.88	65.02 +/− 2.27	0.56
Sex (n) M F	43 34	23 30	0.16
Breslow	1.08+/−0.28	2.65+/−0.34	<0.001
Ulceration (n) Yes No	9 68	12 41	0.09
Mitoses	1.40+/−0.56	3.58+/−0.69	0.016
Satellite lesions (n) Yes No	1 76	3 50	0.16
Lymphovascular invasion (n) Yes No	1 76	3 50	0.16
Perineural invasion (n) Yes No	0 77	3 50	0.03
Lymph node metastases (n) Yes No	8 69	8 45	0.42
In-transit metastases (n) Yes No	1 76	3 50	0.16
Systemic metastases (n) Yes No	1 76	1 52	0.79
